# A comparative profile of methanol extracts of *Allium cepa* and *Allium sativum* in diabetic neuropathy in mice

**DOI:** 10.4103/0974-8490.75460

**Published:** 2010

**Authors:** Abhishek Bhanot, Richa Shri

**Affiliations:** *Amar Shaheed Baba Ajit Singh Jujhar Singh Memorial (ASBASJSM) College of Pharmacy, BELA, Ropar - 1401 11, Punjab, India*; 1 *Pharmacognosy Division, Department of Pharmaceutical Sciences and Drug Research, Punjabi University, Patiala - 1470 02, Punjab, India*

**Keywords:** *Allium cepa*, *Allium sativum*, diabetic neuropathy, oxidative stress

## Abstract

**Introduction:**

Diabetic Neuropathy (DN) is a major microvascular complication of uncontrolled diabetes. This may result from increased oxidative stress that accompanies diabetes. Hence plants with antioxidant action play an important role in management of diabetes and its complications.

**Materials and Methods:**

This study was designed to evaluate preventive as well as curative effect of methanol extracts of outer scales and edible portions of two plants with established antioxidant action - *Allium cepa* and *Allium sativum*, in induced DN in albino mice. Mice were divided into control, diabetic and test extracts treated groups. Test extracts were administered daily at a dose of 200 mg/kg p.o. for 21 days, in the preventive group prior to onset of DN, and in the curative group after the onset of DN. Hyperalgesia and oxidative stress markers were assessed. STZ-diabetic mice showed a significant thermal hyperalgesia (as assessed by the tail-flick test), indicating development of DN.

**Results:**

Treatment with test extracts prevented loss in body weight, decreased plasma glucose level, and significantly ameliorated the hyperalgesia, TBARS, serum nitrite and GSH levels in diabetic mice.

**Conclusion:**

Methanol extract of outer scales of onion has shown most significant improvement; may be due to higher content of phenolic compounds in outer scales of *A. cepa*.

## INTRODUCTION

Diabetes mellitus (DM) is a serious problem in developing as well as developed countries.[1] Uncontrolled diabetes leads to microvascular and macrovascular complications.[2] The most common microvascular complication is neuropathy. It affects 20–30 million people worldwide and is a significant source of morbidity and mortality.[3,4]

The exact pathophysiological mechanism by which nerves are damaged in diabetic neuropathy (DN) is controversial but prolonged hyperglycemia is an accepted primary causative mechanism.[5] Metabolic mechanisms of glucose-induced neurotoxicity include nonenzymatic glycation of proteins with subsequent chemical rearrangements, yielding complex protein adducts known as advanced glycation end products (AGES);[6,7] autooxidation of glucose;[8] increased aldose reductase (AR) activity leading to sorbitol and fructose accumulation, Nicotinamide adenine dinucleotide phosphate (NADP) redox imbalances; alterations in signal transduction;[9–11] activation of protein kinase C,[12] increased *de novo* synthesis of diacylglycerol (DAG) from glucose and inhibition of DAG kinase,[13–15] *hyperglycemia-induced overproduction of reactive free radical molecules leading oxidative stress*,[16,17] and neuroinflammation.[18,19] Oxidative stress in chronic hyperglycemia and in the development of neuropathy has been examined in animal models.[20]

Early detection and control of diabetes and coexisting risk factors for neuropathy (e.g., smoking, alcohol abuse, hypertension) can prevent, delay, or slow the progression of DN.[21–24] Naturally occurring plant contents such as phenolic compounds, carotenoids, ascorbic acid, thiols, and tocopherols have shown antioxidant activity that includes scavenging free radical species, and inhibiting the production of reactive species resulting from normal cell metabolism, thereby preventing damage to lipids, proteins, nucleic acids and subsequent cellular damage and death.[25,26]

*Allium cepa* L. (onion) and *Allium sativum* L. (garlic) are bulbous herbs belonging to family Alliaceae and are commercially cultivated worldwide.[27] Both are widely used food ingredients. *A. cepa*, a potent antioxidant, is one of the richest sources of flavonoids and organosulphur compounds;[28–32] anthrocyanin pigments are reported from red onions.[33] In *A. sativum* a small amount of non-volatile sulphur-containing compounds such as S-allyl cysteine (SAC) and S-allyl mercaptocysteine (SAMC) have significant antioxidant activity (Imai *et al*., 1994). Allixin and organo-selenium compounds are other characteristic major chemical constituents of garlic which exhibit antioxidant effect synergistically with organo-sulphur compounds.[34]

Garlic and onion extracts have been reported to possess anticarcinogenic activity. They are reported to have significant antimutagenic activities in the bladder,[35] brain,[36] breast,[37] colon,[38] lung,[39] ovary,[40] and stomach[41] cancers. Both have antimicrobial activity including antibacterial,[42] antifungal,[43] antiparasitic,[44] and antiviral[45] actions. These also show cardiovascular effect which includes antihypertensive, antiplatelet and antithrombotic action.[46,47] Both possess higher antioxidant level effect.[48,49] Garlic and onion also show immunmodulatory,[50] antidiabetic[51] and neuroprotective[52] activity.

As discussed above, *A. cepa* and *A. sativum* possess a high level of anti-oxidant activity, which is attributed to the flavonoids and organosulphur compounds and have demonstrated antidiabetic effect but the effect of *A. cepa* and *A. sativum* extracts on diabetic neuropathy has not been evaluated. Hence the present study has been designed to investigate the effect of methanol extracts of *A. cepa* and *A. sativum* on diabetic neuropathy in mice.

### Experimental

#### Plant

Bulbs of *A. cepa* L. var. agrifound Dark Red and *A. sativum* were collected from the National Horticulture Research and Development Foundation (NHRDF), Karnal (Haryana), India and *A. cepa* L. var. agrifound Dark Red and *A. sativum* were used in the study identified and authenticated by Dr. K.P.S. Chauhan, Director NHRDF, Karnal (Haryana).

### Preparation of extracts

Extracts were prepared separately from outer scales and edible portion of bulbs. Dried skin (outer scales) of both plants bulbs (30 g) was ground with 90% methanol and kept for 30 min in an ultrasonic bath, then allowed to stand for 24 h at room temperature. Ernatant was taken and filtered. The solvent was removed under reduced pressure at 35-40°C using a rotatory vacuum evaporator and the dried extract was weighed.[53]

An extract of edible portion of onion bulbs (100 g) was prepared. This was sliced into boiling water to inactivate the enzyme allinase. It was then ground with 80% methanol. After vacuum filtration, the filtrate was collected and concentrated on rotatory evaporator at 40-43°C and dried extract was weighed.[54]

Dried edible portion of garlic bulbs (100 g) was extracted with 80% methanol in Soxhlet apparatus for 72 h. After extraction, the solvent was filtered and then evaporated by rotatory evaporator and dried extract was weighed.[55]

### Animals

Swiss albino mice (25-35 g) of either sex were employed in the present study. Animals were housed in institutional animal house under standard conditions, with 12-h light/dark cycle and maintained on standard laboratory diet (Kisan Feeds Ltd., Mumbai, India) and had free access to tap water. The experimental protocol was duly approved by the Institutional Animal Ethical Committee (IAEC) of Punjabi University, Patiala; care of animals was carried out according to the guidelines of the Committee for the Purpose of Control and Supervision of Experiments on Animals (CPCSEA), Ministry of Environment and Forests, Government of India (Reg. No.-107/1999/ CPCSEA).

### Drugs and Reagents

α-napthol, chloral hydrate, ferric chloride, gelatin, iodine, nitric acid, picric acid, potassium iodide, sodium hydroxide, sodium chloride, sulphuric acid and vanillin (Loba Chemie) were used for phytochemical screening of the plant extracts. 1, 1, 3, 3-tetraethoxy propane, thiobarbituric acid, nitroblue tetrazolinium chloride (Sigma aldrich, India) was used for evaluating lipid peroxidation by TBARS, DTNB (Loba Chemie) and trichloroacetic acid was used for evaluating reduced glutathione level and N-naphthylethylenediamine and copper cadmium alloy for evaluating Serum nitrite level. All other chemical reagents used in the study were analytical grade.

### Determination of acute toxicity (LD_50_)

The acute toxicity study on all four extracts of *Allium cepa* and *Allium sativum* was carried out in albino mice. The animals were fasted overnight prior to the experiment. Fixed dose method of CPCSEA was adopted for toxicity studies.[56]

### Experimental protocol

In the present study 12 groups of animals was employed. Each group comprised of 6 mice.

**Table:** 

EXPERIMENTAL	PROTOCOL
↓	↓
To prevent onset of Diabetic Neuropathy Treatment started after 7^th^ day of STZ injection	To treat Diabetic Neuropathy Treatment started after 21^st^ day of STZ injection

### Preventive study

Treatment of test groups was started after seventh day of STZ injection.

### Group I Normal control

It consisted of nondiabetic mice administered with 0.1 N citrate buffer. Body weight of mice and tail immersion test were noted before and after administration of 0.1N citrate buffer on different days i.e. 0, 7^th^, 14^th^, 21^st^ day. Fasting blood glucose was monitored after administration of citrate buffer on 0, 4^th^, 7^th^, 14^th^, 21^st^ days. At the end of the study mice were sacrificed on the 21^st^ day and the brain tissue preserved for further estimation of serum nitrite, glutathione, TBARS levels.

### Group II Diabetic control

Mice were rendered diabetic by single-dose administration of STZ (100 mg\kg i.p.). Different parameters were noted on different days as described in Group I.

### Group III Methanol extract of Outer Scales of *A. cepa*:

Group III comprised of mice administered methanol extract of outer scales of *A. cepa* (200 mg/kg, p.o.).

### Group IV Methanol extract of Edible Portion of *A. cepa*:

Group IV comprised of mice administered methanol extract of edible portion of *A. cepa* (200 mg/kg, p.o.).

### Group V Methanol extract of Outer Scales of *A. sativum*:

Group V comprised of mice administered methanol extract of outer scales of *A. sativum* (200 mg/kg, p.o.).

### Group VI Methanol extract of Edible Portion of *A. sativum*:

Group VI comprised of mice administered methanol extract of outer scales of *A. sativum* (200 mg/kg, p.o.).

The test extracts were administered daily for 14 days to STZ-diabetic mice. The various parameters were evaluated on different days as described in Group I.

### Curative study

Treatment of test groups was started after onset of DN as confirmed by tail immersion test.

### Group VII Normal control

It consisted of nondiabetic mice administered with 0.1 N citrate buffer. Body weight of mice and tail immersion test were noted before and after administration of 0.1N citrate buffer on different days i.e. 0, 7^th^, 14^th^, 21^st^, 28^th^, 35^th^ day. Fasting blood glucose was monitored after administration of citrate buffer on 0, 4^th^, 7^th^, 14^th^, 21^st^, 28^th^, 35^th^ days. At the end of the study mice were sacrificed on the 35^th^ day and the brain tissue preserved for further estimation of serum nitrite, glutathione, TBARS levels.

### Group VIII Diabetic control

Mice were rendered diabetic by single dose administration of STZ (100 mg\kg i.p.). Various parameters were noted on different days as described in Group VI.

### Group IX Methanol extract of Outer Scales of *A. cepa*

roup IX comprised of mice administered methanol extract of outer scales of *A. cepa* (200 mg/kg, p.o.).

### Group X Methanol extract of Edible Portion of *A. cepa*

Group X comprised of mice administered methanol extract of edible portion of *A. cepa* (200 mg/kg, p.o.).

### Group XI Methanol extract of Outer Scales of *A. sativum*

Group V comprised of mice administered methanol extract of outer scales of *A. sativum* (200 mg/kg, p.o.).

### Group XII Methanol extract of Edible Portion of *A. sativum*

Group XII comprised of mice administered methanol extract of outer scales of *A. sativum* (200 mg/kg, p.o.).

The test extracts were administered daily for 14 days in mice with DN i.e. after 21 days of STZ administration. The various parameters were evaluated on different days as described in Group VI.

### Induction of Experimental Diabetes Mellitus and Neuropathy in Mice:

The mice were rendered diabetic by a single intraperitonial injection of streptozotocin (STZ) (100 mg/kg) dissolved in 0.1 N citrate buffer (pH 4.5).[57–60] Blood samples for glucose estimation were obtained from the retro-orbital sinus. Mice with fasting blood glucose level more than 13.9 mmol\L were considered to be diabetic and were included for the study.[61] These diabetic mice were kept for further 21 days for development of DN.[62]

### Evaluation of pharmacological parameters Body Weight

DN is associated with weight loss.[63] The body weight of experimental animals was determined using a commercially available weighing balance, prior to STZ treatment and after the administration of STZ. Body weight was measured weekly.

### Behavioral study

The behavioral tests were performed according to the experimental protocol. The experiment was performed between 9:00 am to 5:00 pm in the laboratory at standard optimum conditions.

### Tail immersion test (Warm water)

Tails of mice were immersed in a warm water bath (52.5 ± 0.5°C) until tail withdrawal (flicking response) or signs of struggle were observed (cutoff time 12 sec). Shortening of the tail withdrawal time indicates hyperalgesia and is attributed to central mechanisms.[64,65]

### Biochemical parameters

Serum and plasma separation Blood was withdrawn retro-orbitally with the help of microcapillaries. The collected blood was kept for 30 min at room temperature. Serum and plasma was separated out by centrifugation. The plasma and serum was used for glucose and serum nitrite estimation.

### Estimation of Blood Glucose

Blood glucose level was estimated colorimetrically at 505 nm by Glucose Oxidase-Peroxidase (GOD-POD) method using a commercially available kit (Transasia Bio Medicals Ltd. Daman).[66]

### Estimation of Serum Nitrite

To 100 µl of serum sample in test tube, 400 µl carbonate buffer (pH 9.0) was added and followed by addition of approximately 0.15 g of copper cadmium alloy fillings. The test tube was incubated at room temperature for 1 h with intermittent shaking. The reaction was stopped by addition of 100 µL of 0.35 M sodium hydroxide and 400 µL of 120 mM zinc sulphate solution (in distilled water) under vortex mixing. The solution was allowed to stand for 10 min and centrifuged at 4000 rpm for 10 min. 500µL of the clear supernatant was transferred to another test tube and 250 µL of 1% sulphanilamide (in 3N HCL) and 250µL of 0.1% N-naphthylethylenediamine (in distilled water) were added. After 10 min, the absorbance was noted spectrophotometerically at 545 nm against suitably prepared blank solution (100 µL of distilled water was used instead of serum). A standard curve (0.1-10 µM) using sodium nitrite was plotted to calculate the concentration of nitrite.[67]

### Tissue homogenate preparation

Brain was homogenized in 0.1 M Tris HCl buffer (pH 7.4, 10% w/v) using Pestle motors. The clear supernatant of homogenate was used to estimate total protein content, TBARS and reduced glutathione after centrifugation at 800 g for 10 min.

### Estimation of Thiobarbituric Acid Reactive Substance (TBARS)

The quantitative measurement of thiobarbituric acid reactive substances (TBARS), an index of lipid peroxidation in the brain was performed according to the method of Ohkawa *et al*.[68] The absorbance of developed pink color was measured spectrophotometrically at 532 nm.

### 2.8.7 Estimation of Brain protein content

The brain protein content was determined by the Lowry method.[69] The protein content was determined spectrophotometrically at 750 nm and expressed as mg per g.

### Estimation of reduced glutathione

The reduced glutathione (GSH) content in brain was estimated using the method of Beutler *et al*.[70] and absorbance was noted spectrophotometrically at 412 nm.

### Statistical Analysis

All the results were expressed as mean ± standard error of means (S.E.M). The data of behavioral results was statistically analyzed by two-way analysis of variance (ANOVA) followed by Bonferonni’s post test by using Graph pad prism Version-5.0 software. The data of biochemical results was statistically analyzed by one-way ANOVA followed by Tukey’s multiple range tests. *p*-value < 0.001 was considered to be statistically significant.

## RESULTS

### Determination of acute toxicity (LD_50_)

The LD_50_ was determined by using fixed dose method as recommended by CPCSEA, OECD guideline no. 420. There was no death observed in all four extracts (i.e. methanol extract of both outer scales and edible portion of *A. cepa* and *A. sativum*) up to 24 h.

### Yield of methanol extracts of Onion and Garlic

Methanol extracts of different parts of the two plants were prepared and yields of different extracts are reported in Table 1.

**Table 1:** Yield of methanol extracts of *A. cepa* and *A. sativum*

Plants	Outer scales	Edible portion
*A. cepa*	6.7%	6.8%
*A. sativum*	4.71%	7.0%

### Effect of plant extracts in Diabetic Neuropathy Preventive group

In this group experimental animals with diabetes but prior to onset of DN were administered the test extracts in order to observe whether these prevented onset of DN.

### Effect of Methanol extracts of *A. cepa* and *A. sativum* on blood glucose level

DM is characterized by increase in the level of blood glucose when compared to normal control. Administration of different extracts once daily for 14 days starting from seventh day of STZ administration produced varied effect on blood glucose of experimental animals [Table 2]. Administration of methanol extract of outer scales of *A. cepa* (200 mg/kg) produceda significant decrease in plasma blood glucose level and methanol extract of outer scales of *A. sativum* (200 mg/kg) did not show any significant change in the plasma blood glucose level. While the administration of methanol extract of edible portions of *A. cepa* and *A. sativum* (200 mg/kg) produceda significant decrease in plasma blood glucose level.

**Table 2:** Effect of methanol extract on Blood Glucose levels (in mmol\L) (Preventive Group)

Groups	Day 1	Day 4	Day 7	Day 14	Day 21
Normal control	5.5±0.15	5.6±0.16	5.4±0.16	5.5±0.14	5.6±0.14
Diabetic control	5.4±0.16	17.4±0.51^a^	17.9±0.52^a^	18.3±0.5^a^	18.8±0.5^a^
Onion outer scale	5.4±0.12	16.6±0.23	17.0±0.23	15.6±0.2^b^	15.1±0.19^b^
Onion edi. Portion	5.4±0.15	16.6±0.21	17.0±0.15	16.5±0.15^b^	15.9±0.13^b^
Garlic outer scale	5.6±0.15	16.6±0.22	17.0±0.18	17.6±0.21	18.1±0.23
Garlic edi. Portion	5.6±0.15	16.7±0.17	17.0±0.16	16.6±0.16^b^	16.1±0.12^b^

Values are mean ± SEM, n=6, a=*P*<0.001 vs. normal control group, b=*P*<0.001 vs. diabetic control group

### Effect of Methanol extracts of *A. cepa* and *A. sativum* on weight variation:

DM results in significant decrease in the body weight when compared to normal control. Administration of different extracts once daily for 14 days starting from seventh day of STZ administration produced varied effect on weight loss of experimental animals [Table 3]. Administration of methanol extract of outer scales of *A. cepa* (200 mg/kg) prevented loss in body weight and methanol extract of outer scales of *A. sativum* (200 mg/kg) did not show any significant change in body weight of mice. Administration of methanol extract of edible portions of *A. cepa* and *A. sativum* (200 mg/kg) did not show any significant change in body weight of mice.

**Table 3:** Effect of methanol extract on Weight Variation (in grams) (Preventive Group)

Groups	Day 0	Day 7	Day 14	Day 21
Normal Control	28±0.78	29.1±0.79	30.6±0.73	32.1±0.64
Diabetic Control	30.6±0.9	27.0±0.78	24.6±0.77^a^	22.6±0.77^a^
Onion outer scale	30.6±1.31	25.6±1.28	25.5±1.41	28.3±1.28^b^
Garlic outer scale	30.3±1.1	26.6±0.9	25.0±0.81	23.0±0.81
Onion edi. portion	30.3±1.45	25.6±1.45	25.3±1.47	26.6±1.54
Garlic edi. portion	30.3±0.87	26.0±0.94	25.3±0.77	26.0±0.94

Values are mean ± SEM, n=6, a=*P*<0.001 vs. normal control group, b=*P*<0.001 vs. diabetic control group

### Effect of Methanol extracts of *A. cepa* and *A. sativum* on Tail Immersion latency time

DN resulted in significant decrease in the tail immersion latency time when compared to normal control. Administration of different extracts once daily for 14 days starting from the seventh day of STZ administration produced varied effect on the tail immersion latency time of experimental animals [Table 4]. Administration of methanol extract of outer scales of *A. cepa* (200 mg/kg) produced a significantincrease in tail immersion latency time and methanol extract of outer scales of *A. sativum* (200 mg/kg) did not show any significant change in tail immersion latency time. Administration of methanol extract of edible portions *A. cepa* and *A. sativum* (200 mg/kg) produced a significantincrease in tail immersion latency time.

**Table 4:** Effect of methanol extract on Tail immersion latency time (in seconds) (Preventive Group)

Groups	Day 1	Day 4	Day 7	Day 14	Day 21
Normal control	8.4±0.21	8.4±.28	8.4±0.29	8.3±0.25	8.2±0.29
Diabetic control	8.3±0.2	7.6±.19	6.0±0.12^a^	4.6±0.09^a^	3.9±0.09^a^
Onion outer scale	8.3±0.24	7.6±.21	6.2±0.07	6.4±0.08^b^	6.5±0.07^b^
Onion edi. portion	8.3±0.22	7.5±.22	6.3±0.23	6.0±0.23^b^	5.7±0.22^b^
Garlic outer scale	8.2±0.22	7.4±.21	6.2±0.23	5.4±0.23	4.3±0.22
Garlic edi. portion	8.3±0.27	7.5±.23	6.4±0.2	5.9±0.2b*	5.5±0.19^b^

Values are mean ± SEM, n=6,

a = *P*<0.001 vs. normal control group,

b = *P*<0.001 vs. diabetic control group,

b* = *P*<0.01 vs. diabetic control group

### Effect of Methanol extracts of *A. cepa* and *A. sativum* on Thiobarbituric Acid Reactive Substances (TBARS) level

**Figure 1:**
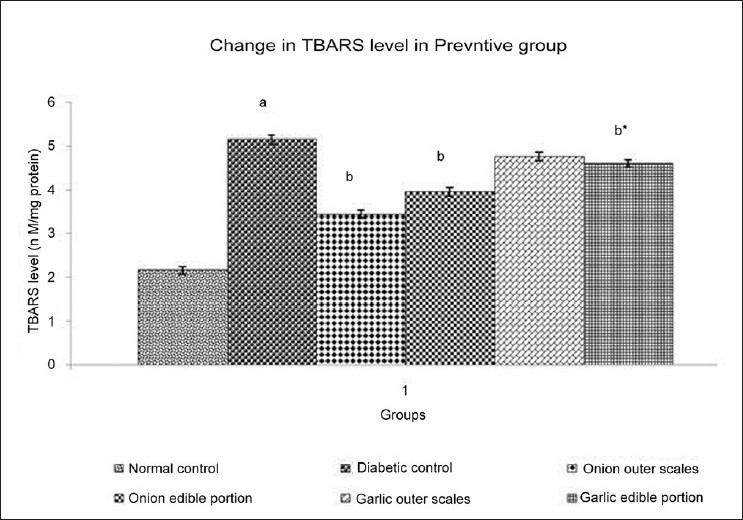
Effect of Methanol Extract of Outer scales and Edible Portions of *Allium cepa* and *Allium sativum* on Thiobarbituric Acid Reactive Substances (TBARS) in Diabetic mice. Values are mean ± SEM, n=6, ^a^ = *P*<0.001 vs. normal control group, ^b^ = *P* < 0.001 vs. diabetic control group, ^b*^ = *P* < 0.01 vs. diabetic control group

Experimental animals with DM showed significant increase in TBARS level when compared to normal control. Administration of different extracts once daily for 14 days starting from the seventh day of STZ administration produced varied effect on the TBARS level of experimental animals [Figure 1]. Administration of methanol extract of outer scales of *A. cepa* (200 mg/kg) produced a significant decrease in TBARS level and methanol extract of outer scales of *A. sativum* (200 mg/kg) did not show any significant change in TBARS level. Administration of methanol extract of edible portions of *A. cepa* and *A. sativum* (200 mg/kg) produced a significant decrease in TBARS level.

### Effect of Methanol extracts of *A. cepa* and *A. sativum* on GSH level

**Figure 2:**
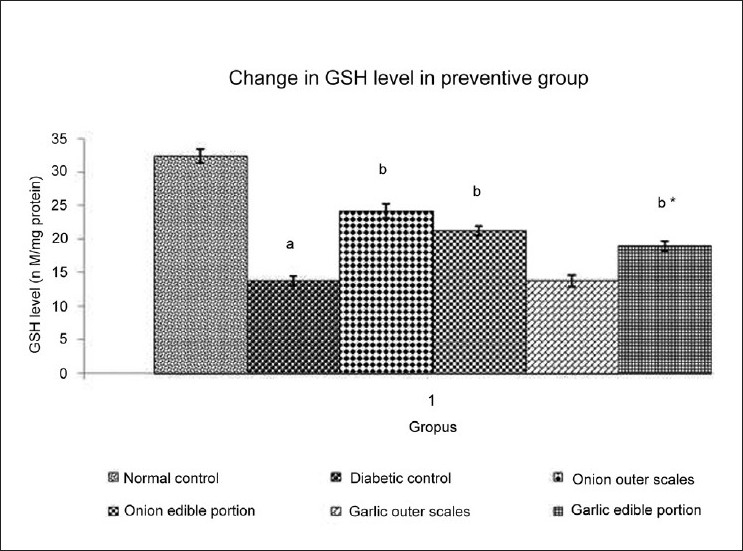
Effect of Methanol Extract of Outer scales and Edible Portions of *Allium cepa* and *Allium sativum* on the GSH level in Diabetic mice Values are mean ± SEM, n=6, ^a^ = *P*<0.001 vs. normal control group, ^b^ = *P* < 0.001 vs. diabetic control group, ^b*^ = *P* < 0.01 vs. diabetic control group

DM resulted in significant decrease in the level of reduced glutathione (GSH) when compared to normal control. Administration of different extracts once daily for 14 days starting from the seventh day of STZ administration produced varied effect on reduced GSH level of experimental animals [Figure 2]. Administration of methanol extract of outer scales of *A. cepa* (200 mg/kg) produced a significantincrease in GSH level and methanol extract of outer scales of *A. sativum* (200 mg/kg) did not show any significant change in GSH level. Administration of methanol extract of edible portions of *A. cepa* and *A. sativum* (200 mg/kg) produced a significantincrease in GSH level.

### Effect of Methanol extracts of *A. cepa* and *A. sativum* on Serum Nitrite level

**Figure 3:**
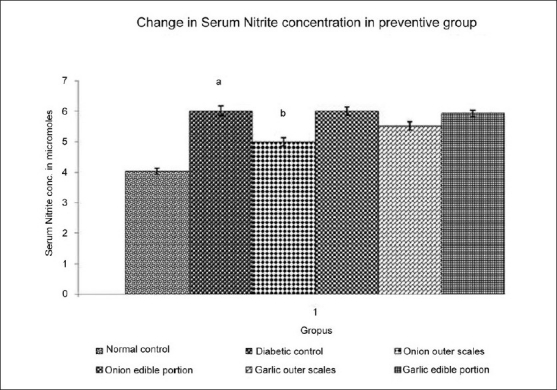
Effect of Methanol Extract of Outer scales and Edible Portions of *Allium cepa* and *Allium sativum* on the Serum Nitrite level in Diabetic mice Values are mean ± SEM, n=6, ^a^ = *P* < 0.001 vs. normal control group, ^b^ = *P* < 0.001 vs. diabetic control group

DM resulted in a significant increase in the level of serum nitrite when compared to normal control. Administration of different extracts once daily for 14 days starting from the seventh day of STZ administration produced varied effect on the serum nitrite level of experimental animals [Figure 3]. Administration of methanol extract of outer scales of *A. cepa* (200 mg/kg) produced a significantdecrease in the serum nitrite level and methanol extract of outer scales of *A. sativum* (200 mg/kg) did not show any significant change in the serum nitrite level. Administration of methanol extract of edible portions of *A. cepa* and *A. sativum* (200 mg/kg) did not show any significant change in the serum nitrite level.

### Result in Curative Group

In this group diabetic animals in whom onset of DN was observed were administered test extracts in order to determine if these could reverse the symptoms of DN.

### Effect of Methanol extracts of *A. cepa* and *A. sativum* on blood glucose level in diabetic neuropathy

DM is characterized by increase in the levels of blood glucose when compared to normal control. Administration of different extracts once daily for 14 days starting from the 21^st^ day of STZ administration produced varied effect on blood glucose of experimental animals [Table 5]. Administration of methanol extract of outer scales of *A. cepa* (200 mg/kg) produced a significantdecrease in blood glucose level; methanol extract of outer scales of *A. sativum* (200 mg/kg) did not show any significant change in blood glucose level. Administration of methanol extract of edible portions of *A. cepa* and *A. sativum* (200 mg/kg) produced a significantdecrease in blood glucose level.

**Table 5:** Effect of methanol extract on Blood Glucose levels (in mmol\L) (Curative Group)

Groups	Day 1	Day 4	Day 7	Day14	Day 21	Day 28	Day 35
Normal control	5.5±0.15	5.6±0.16	5.4±0.16	5.5±0.14	5.6±0.14	5.5±0.16	5.6±0.1
Diabetic control	5.4±0.16	17.4±0.51^a^	17.9±0.52^a^	18.3±0.5^a^	18.8±0.5^a^	19.5±0.5 a	20.1±0.45^a^
Onion outer scale	5.4±0.14	16.9±0.21	17.3±0.2	17.7±0.19	18.1±0.17	16.5±0.13 b	15.8±0.12^b^
Onion edi. Portion	5.4±0.15	16.8±0.15	17.2±0.13	17.7±0.12	18.1±0.12	17.1±0.1 b	16.3±0.07^b^
Garlic outer scale	5.5±0.13	16.8±0.16	17.2±0.16	17.6±0.12	18.1±0.09	18.8±0.14	19.2±0.15
Garlic edi. Portion	5.6±0.14	16.7±0.18	17.2±0.17	17.6±0.15	18.0±0.14	17.7±0.14 b	17.3±0.15^b^

Values are mean ± SEM, n=6,

a = *P* < 0.001 vs. normal control group,

b = *P* < 0.001 vs. diabetic control group

### Effect of Methanol extracts of *A. cepa* and *A. sativum* on weight variation in diabetic neuropathy

DM resulted in significant decrease in the body weight when compared to normal control. In diabetic animals with neuropathy body weight continued to decrease. Administration of different extracts once daily for 14 days starting from the 21^st^ day of STZ administration produced varied effect on the weight loss of experimental animals [Table 6]. Administration of methanol extract of outer scales of *A. cepa* (200 mg/kg) produced a significantincrease in body weight; methanol extract of outer scales of *A. sativum* (200 mg/kg) did not show any significant change in body weight. Administration of methanol extract of edible portions of *A. cepa* and *A. sativum* (200 mg/kg) produced a significantincrease in body weight.

**Table 6:** Effect of methanol extract on Weight Variation (in grams) (Curative Group)

Groups	Day 1	Day 7	Day 14	Day 21	Day 28	Day 35
Normal Control	28.0±0.78	29.1±0.79	30.6±0.73	32.1±0.64	33.5±0.51	34.3±0.38
Diabetic Control	31.0±1.39	27.0±.078	24.6±0.77^a^	22.6±0.77^a^	20.8±0.68^a^	19.3±0.56^a^
Onion outer scale	30.6±1.22	26.0±1.05	23.6±0.99	22.3±0.73	24.8±0.86	27.0±0.74^b^
Garlic outer scale	30.0±1.05	26.0±1.05	24.0±1.05	22.5±0.99	22.0±1.05	21.0±1.05b*
Onion edi. portion	30.3±1.1	26.6±0.9	24.6±0.9	23.1±0.95	24.5±0.96	25.8±1.01
Garlic edi. portion	27.3±1.7	23.0±1.4	20.8±1.3	19.6±0.87	21.8±1.01	23.5±1.3b**

Values are mean ± SEM, n=6,

a = *P* < 0.001 vs. normal control group,

b = *P* < 0.001 vs. diabetic control group,

b* = *P* <0.01 vs. diabetic control group,

b** = *P* < 0.05 vs. diabetic control group

### Effect of Methanol extracts of *A. cepa* and *A. sativum* on tail immersion latency time in diabetic neuropathy

DN resulted in significant decrease in the tail immersion time when compared to normal control. Administration of different extracts once daily for 14 days starting from the 21^st^ day of STZ administration produced varied effect on the tail immersion latency time of experimental animals [Table 7]. Administration of methanol extract of outer scales of *A. cepa* (200 mg/kg) produced a significantincrease in tail immersion latency time; methanol extract of outer scales of *A. sativum* (200 mg/kg) did not show any significant change in tail immersion latency time. Administration of methanol extract of edible portions of *A. cepa* and *A. sativum* (200 mg/kg) produced a significantincrease in tail immersion latency time.

**Table 7:** Effect of methanol extract on Tail immersion latency time (in seconds) (Curative Group)

Groups	Day 1	Day 4	Day 7	Day 14	Day 21	Day 28	Day 35
Normal control	8.4±0.21	8.4±0.28	8.4±0.29	8.3±0.25	8.2±0.29	8.4±0.29	8.4±0.29
Diabetic control	8.3±0.2	7.6±0.19	6.0±0.12^a^	4.6±0.09^a^	3.9±0.09^a^	2.1±0.15^a^	0.7±0.12^a^
Onion outer scales	8.3±0.25	7.7±0.26	6.4±0.21	4.8±0.17	3.7±0.17	3.8±0.16^b^	4.0±0.15^b^
Onion edi. Portion	8.3±0.2	7.5±0.18	6.3±0.2	4.8±0.16	3.9±0.14	3.6±0.15^b^	3.3±0.15^b^
Garlic outer scales	8.3±0.23	7.4±0.17	6.4±0.12	4.9±0.09	3.9±0.12	2.5±0.15	1.1±0.07
Garlic edi. Portion	8.3±0.21	7.5±0.27	6.5±0.23	4.8±0.12	3.7±0.13	3.3±0.14^b^	2.9±0.15^b^

Values are mean ± SEM, n=6,

a = *P* < 0.001 vs. normal control group,

b = *P* < 0.001 vs. diabetic control group

### Effect of Methanol extracts of *A. cepa* and *A. sativum* on Thiobarbituric Acid Reactive Substances (TBARS) level in diabetic neuropathy

**Figure 4:**
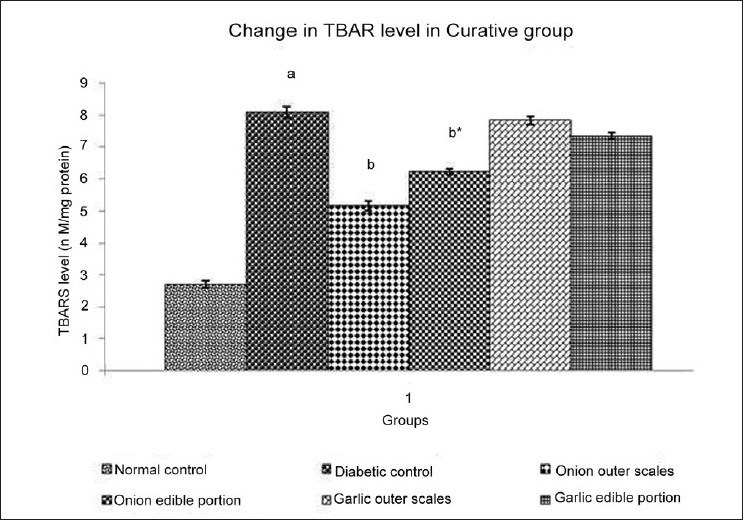
Effect of Methanol Extract of Outer scales and Edible Portions of *Allium cepa* and *Allium sativum* on TBARS level in mice with Diabetic Neuropathy. Values are mean ± SEM, n=6, ^a^ = *P*<0.001 vs. normal control group, ^b^ = *P* < 0.001 vs. diabetic control group, ^b*^ = *P* < 0.01 vs. diabetic control group

Experimental animals with DN showed significant increase in TBARS level when compared to normal control. Administration of different extracts once daily for 14 days starting from the 21^st^ day of STZ administration produced varied effect on the TBARS level of experimental animals [Figure 4]. Administration of methanol extract of outer scales of *A. cepa* (200 mg/kg) produced a significantdecrease in TBARS level and *A. sativum* (200 mg/kg) did not show any significant change in TBARS level. Administration of methanol extract of edible portions of *A. cepa* and *A. sativum* (200 mg/kg) produced a significantdecrease in TBARS level.

### Effect of Methanol extracts of *A. cepa* and *A. sativum* on reduced glutathione level in diabetic neuropathy

**Figure 5:**
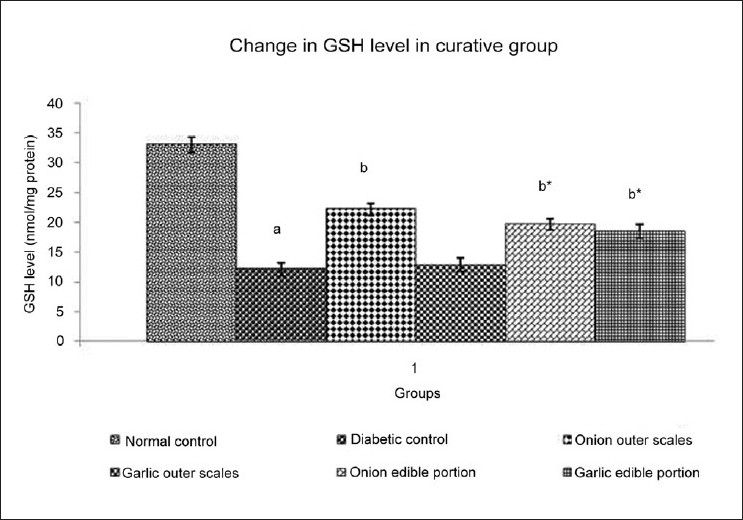
Effect of Methanol Extract of Outer scales and Edible Portions of *Allium cepa* and *Allium sativum* on TBARS level in mice with Diabetic Neuropathy. Values are mean ± SEM, n=6, ^a^ = *P*<0.001 vs. normal control group, ^b^ = *P* < 0.001 vs. diabetic control group, ^b*^ = *P* < 0.01 vs. diabetic control group

DN resulted in a significant decrease in the level of reduced glutathione (GSH) when compared to normal control. Administration of different extracts once daily for 14 days starting from the 21^st^ day of STZ administration produced varied effect on the GSH level of experimental animals [Figure 5]. Methanol extract of the outer scales of *A. cepa* (200 mg/kg) produced a significantincrease in the GSH level; methanol extract of outer scales of *A. sativum* (200 mg/kg) did not show any significant change in the GSH level. Administration of methanol extract of edible portions of *A. cepa* and *A. sativum* (200 mg/kg) produced a significantincrease in the GSH level.

### Effect of Methanol extracts of *A. cepa* and *A. sativum* on the serum nitrite level in diabetic neuropathy

**Figure 6:**
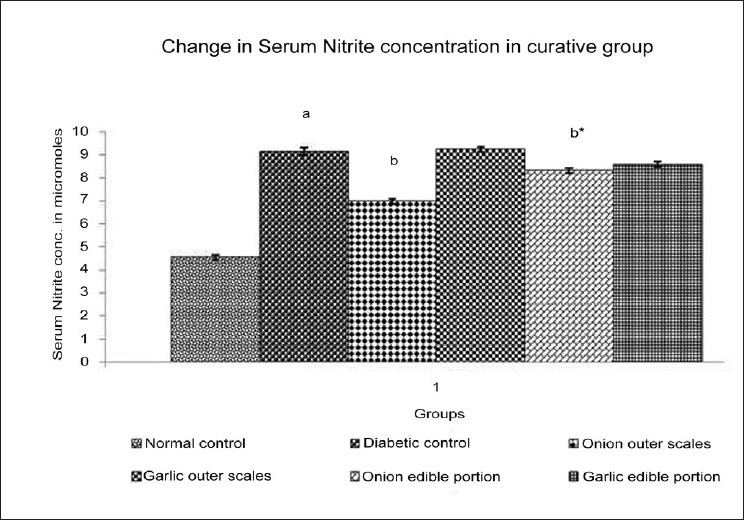
Effect of Methanol Extract of Outer scales and Edible Portions of *Allium cepa* and *Allium sativum* on the Serum Nitrite level in mice with Diabetic Neuropathy. Values are mean ± SEM, n=6, ^a^ = *P*<0.001 vs. normal control group, ^b^ = *P* < 0.001 vs. diabetic control group, ^b*^ = *P* < 0.01 vs. diabetic control group

DN resulted in a significant increase in the level of serum nitrite when compared to normal control. Administration of different extracts once daily for 14 days starting from the 21^st^ day of STZ administration produced varied effect on the serum nitrite level of experimental animals [Figure 6]. Methanol extract of outer scales of *A. cepa* (200 mg/kg) produced a significantdecrease in the serum nitrite level; *A. sativum* (200 mg/kg) did not show any significant change in the serum nitrite level. Methanol extract of edible portions of *A. cepa* (200 mg/kg) produced a significantdecrease in the serum nitrite level and *A. sativum* (200 mg/kg) did not show any significant change in the serum nitrite level.

## DISCUSSION

STZ-induced diabetes is characterized by uniform hyperglycemia. STZ selectively destroys pancreatic β cells and destruction of β cells is associated with deficiency of insulin and induction of insulin-dependent diabetes mellitus (IDDM). STZ-induced β cell destruction is due to fragmentation of DNA in pancreatic β cells[71,72] that may be mediated by alkylation of DNA.[73,74] A single dose of streptozotocin (100 mg\kg, i.p.) is well reported to induce IDDM in mice.[57–60]

Mice with glucose concentration of more than 13.9 mmol\L[61] were employed in the study. Uncontrolled hyperglycemia for two weeks is reported to produce significant hyperalgesia, a characteristic symptom of diabetic neuropathy in mice.[75] The experimental animals were kept for 21 days to provide sufficient time for hyperglycemia to effect the pain perception. Tail immersion latency time was monitored for the assessment of degree of hyperalgesia.

Experimental diabetes mellitus-induced hyperglycemia produced a significant decrease in tail flick latency time. The decrease in tail flick latency was directly related to the duration of hyperglycemia. Hyperalgesia was more pronounced after the 21^st^ day of the experimental protocol. Weight loss is not due to neuropathy nevertheless often accompanies DN. Free radicals promote lipid peroxidation,[76] which results in the alteration of permeability and fluidity of membranes.[77] Reactive oxygen species (ROS) produces malondialdehyde (MDA), an end product of lipid peroxidation. MDA reacts with thiobarbituric acid (TBA) and is thus estimated as thiobarbituric acid reactive substances (TBARS) in cells.[78,79] In DN the level of glutathione decreases. Impaired glutathione metabolism would be expected to weaken the defense against oxidative stress.[80] Nitric oxide is converted to nitrite in the presence of oxygen, water and hemoglobin. Therefore, the serum nitrite level was estimated by Greiss reaction, which served as an indirect index of nitric oxide production.

Studies with antioxidants support the role of plants in DN.[59] As reported earlier, onions (*Allium cepa*) and garlic (*Allium sativum*) possess a high level of antioxidant activity.[34,81] Anthocyanins pigment[33] and organosulphur compounds such as thiosulphinates and cepaenes[28,30] in onion and non-volatile sulphur compounds like S-allyl cysteine, S-allyl mercepto cysteine and organo-selenium compounds in garlic are potent antioxidants.

Recent studies have shown that antioxidant compounds quercetin and its derivatives were isolated from the methanol extract of the dry outer scales of onion (*Allium cepa*. L).[53] It is observed that red onions possess a high amount of phenolic compounds i.e. quercetin in comparison to white onion and garlic.[82] This supports the fact that in this investigation the most significant improvement in DN was observed with the extract of the outer scales of onion (variety dark red).

## CONCLUSION

The results of the present study show that the extract of the outer scale of onion and the edible portion of both onion and garlic provided significant protection in DN in both preventive and curative groups. The methanol extract of the outer scales of onion has shown a most significant effect which may be due to the presence of higher quantities of phenolic compounds.
